# Physical capacity and psychological mood in association with self-reported work ability in vibration-exposed patients with hand symptoms

**DOI:** 10.1186/1745-6673-7-22

**Published:** 2012-11-09

**Authors:** Maria Edlund, Lars Gerhardsson, Mats Hagberg

**Affiliations:** 1 Department of Occupational and Environmental Medicine, University of Gothenburg, Gothenburg, Sweden

**Keywords:** Quantitative test, Work Ability Index, WAI, Mental condition, HADS, Vibration, Hand symptoms, Grip strength, Manual dexterity

## Abstract

**Background:**

The aim of this study was to investigate whether self-reports of work ability correlated to the results of quantitative tests measuring physical capacity and a questionnaire assessing psychological mood in vibration-exposed patients with hand symptoms.

**Methods:**

The participants comprised 47 patients (36 men and eleven women) with exposure to hand vibration and vascular and/or neurological symptoms in the hands. They performed several quantitative tests (manual dexterity, hand grip strength, finger strength) and completed the Work Ability Index (WAI) and Hospital Anxiety and Depression Scale (HADS) questionnaires.

**Results:**

Correlation analysis revealed statistically significant associations between the WAI results, the HADS indices, hand grip and finger strength, and manual dexterity measured using the Purdue Pegboard^®^. Multiple regression analysis revealed age and HADS indices as the strongest predictors of work ability.

**Conclusions:**

The patient’s age and psychological mood may be stronger predictors of work ability compared with results from tests measuring physical capacity of the hands in vibration-exposed patients with hand symptoms. When using the WAI as an instrument for assessing work ability in these patients, health care providers need to be more aware of the impact of the psychological mood.

## Background

Hand-arm symptoms causing dysfunction are common in workers using vibrating hand-held tools [1-4]. The condition is referred to as hand-arm vibration syndrome (HAVS) and has been described as a complex and potentially chronic disabling disorder [5]. The syndrome has become one of the most commonly described diseases in the industrialized world [6] and in an effort to assess the severity of the symptoms, a classification system of staging the sensorineural and vascular symptoms and signs has been developed, the Stockholm Workshop Scale (SWS) [7]. This scale is partly based on quantitative testing of different sensory modalities involving methods that have been criticized for not being standardized or validated and further for not including assessment of functioning or physical disabilities [8].

The complexity and the consequences of HAVS make it important to increase the knowledge of patients with HAVS. It may be useful to gain insight into associations between many different variables that are relevant in a multidimensional perspective on health and functioning of these affected workers. A more integrated view on these workers’ health may be advantageous, with change of focus from symptoms and staging to a wider perspective on health including functioning and work ability [9]. Poole et al. report a relationship between self-reported upper limb disability and quantitative tests in patients with HAVS, using the Disability of the Arm, Shoulder and Hand (DASH) questionnaire [10]. Cederlund et al. performed a study on problems with activities of daily living (ADL), correlated to test results, in a similar group of affected workers [11]. Another study by the same author indicated lower subjective well-being in workers with more severe HAVS symptoms [12]. With these studies a trend of shifting focus has commenced but the aspect of work ability is missing.

Multiple tests of physical capacity and also psychological mood are useful in assessing health of affected workers to gain a more complete picture when giving advice in daily life, but also concerning their work situation and rehabilitation. A similar perspective can be found in the International Classification of Functioning, Disability and Health (ICF) model [13]. Measures of an individual’s functional loss may help in keeping them in appropriate employment [10]. In this assessment process it may be of interest to broaden the perspective also by regarding the individual’s work ability when trying to assess the injury and not only to focus on staging the symptoms. One method of doing this is to use the Work Ability Index (WAI), [11], based on self-reports from the individual, to assess work ability and resources in relation to work demands. Therefore, in order to gain a new perspective on workers’ health and functioning, the aim of this study was to investigate whether work ability correlated to the results of multiple tests, i.e. physical capacity and psychological mood (anxiety and depression), in vibration-exposed patients with hand symptoms.

## Methods

### Subjects

Patients (n = 71) with diagnosed permanent vascular symptoms (white fingers) and/or neurological symptoms in the hands were invited to participate in the study. All had been referred to the Department of Occupational Medicine, Sahlgrenska University Hospital, Gothenburg, Sweden, during the past approximately 5 years with suspected vibration injury and all were manual workers exposed to hand vibrations from hand-held vibrating tools during work.

The patients were initially contacted by letter giving information on the study and its purpose. Shortly thereafter they were contacted by telephone and those who agreed to participate (n = 47) were booked for a medical examination at the hospital where they would also perform the different tests and complete the questionnaires. All participants provided signed written consent before the testing started.

Participants were asked to refrain from smoking or other nicotine use for 1 hour before the testing, and to avoid any exposure to vibration on the day of testing. The reason for this was to minimize negative effects from nicotine and vibration exposure such as constriction of blood vessels and to optimize conditions for physical performance. The duration of the testing and interview was about 3 hours. Each participant was given monetary compensation of SEK 760.

The study was approved by the ethical committee at the University of Gothenburg, and performed in accordance with the ethical standards laid down in the 1964 Declaration of Helsinki.

### Medical assessment

A standard medical assessment, including measurement of blood pressure and a hand and upper extremity examination, was performed by an experienced occupational medicine physician. Symptoms and signs in the hands were staged according to the Stockholm Workshop Scale (SWS). The scale is divided in a sensorineural staging (from stage 0SN to 3SN) and a vascular staging (from 0 to 4) where stage 0 refers to normal, stage 1 is graded as mild, stage 2 as moderate, stage 3 as severe and stage 4 as very severe [7,14]. The patients were also asked to provide a detailed description of their hand vibration exposure (and of the tools they used the most frequently), in hours per day or week and number of years. A computerized medical record was created for each participant.

### Questionnaires

A study nurse and a clinical laboratory technician conducted the different tests and checked that all questionnaires were correctly completed.

#### Work ability

Work ability was measured using the work ability index (WAI). The WAI is an instrument for assessing an individual’s work ability and resources in relation to work demands in health examinations and workplace surveys [15]. The WAI consists of seven dimensions which are used to calculate a total score ranging from 7 to 49 [16,17], which is classified into poor (7–27), moderate (28–36), good (37–43), or excellent (44–49) work ability. The content of the different dimensions has been previously described by Ilmarinen [15].

#### The hospital anxiety and depression scale

The Hospital Anxiety and Depression Scale or HADS [18] is a questionnaire on depression and anxiety disorders (HADS D and HADS A) that is commonly used in clinical practice and research for screening and grading depression and anxiety. The validity of the Hospital Anxiety and Depression Scale has been reviewed by Bjelland et al. [19] who conclude that HADS performs well in screening for caseness of anxiety disorders and depression in patients from non-psychiatric clinics. When HADS was used in primary care populations the test for sensitivity and specificity showed that a score of +8 was the optimal cut-off for psychiatric morbidity [19].

### Description of quantitative tests

#### Hand-grip strength

The maximal grip strength of the hand was measured by a Baseline® Hydraulic Hand Dynamometer (Fabrication Enterprises Incorporated, New York, NY, USA) according to procedures recommended by the manufacturer. The participant was positioned with their elbow at a 90-degree angle and the wrist in a neutral position with the underarm supported by the table. They were then instructed to grasp the handle three times with each hand at 10-second intervals, starting with the dominant hand [20]. Each measurement was recorded in kg, corresponding to the force required to lift 1 kg on the surface of the earth, i.e. 9.8 Newton. In other words, a measurement of 50 kg corresponds approximately to a force of 500 N. The mean of the three measurements was calculated.

#### Finger-grip strength

The key grip (pinch key) and the three-digit pinch (pinch 3-chuck) were used to measure finger strength, with the aid of a hydraulic pinch gauge (PG-60) (North Coast Medical, San José, CA, USA) [20]. The participant was positioned with the instrument in their hand and asked to pinch three times with their fingers at 10-second intervals. The mean of the three measurements was calculated.

#### Manual dexterity

The Purdue Pegboard^®^ (Model 32020) (Lafayette Instrument Company, Lafayette, IN, USA) was used to measure manual dexterity. The participant was asked to place as many pegs as possible in a vertical row, starting with their dominant hand, then the other hand and, finally, both hands. The test result was based on the total number of pegs moved in a 30-second period [21].

### Data analysis

All statistical analyses were performed using version 8.0.2 of the JMP® software package (SAS Institute Inc., Cary, NC, USA).

#### SWS staging

The sensorineural and vascular components of SWS staging were combined in order to dichotomize the SWS staging into one group with mild symptoms and another group with more severe symptoms. The dichotomized variable was expressed as 0 if both stages were < 2, and as 1 if any stage was ≥ 2. This cut-off reflects the recommendations to avoid hand arm vibration exposure if stage 2 (more severe symptoms) or above is present [5]. This variable was then used in the multiple regression model.

#### Correlation analysis

Correlation analysis was used to examine associations between the WAI and the variables of each test. The HADS indices were log transformed before statistical analysis. The rest of the data were consistent with normal distribution, and Pearson correlation was used.

#### Multiple regression analysis

The results for each test with statistically significant p-values were entered into a multiple linear regression model along with the additional independent factors age and the derived dichotomized SWS variable. The multiple regression method of backward selection was used. In the case of two independent variables being highly linearly correlated (i.e. multicollinearity) only one of the variables was entered into the model. The assumption for normal distribution was tested for by using residuals and predicted values.

## Results

A total of 47 individuals (36 men and 11 women) with vascular and/or neurological symptoms in the hands agreed to participate in the study. Most of those declining participation in the study had moved, were unable to be reached or considered themselves to be living too far away.

All participants were able to state duration of exposure to hand-arm vibrating tools. The average exposure time was 20 years with a range of 3–45 years. Altogether 32 responded that they were currently exposed and their average exposure time was 19 years with a range of 3–45 years. (The majority of formerly exposed participants were involuntarily unemployed and four subjects above the age of 65 had recently retired). The average age of the patients was 49 years with a range of 24–69 years.

Mean values and standard deviation of the different test results of physical capacity and mood measurements are presented by work ability level in Table 1. The work ability level is divided into poor (n = 14), moderate (n = 14), good (n = 14) and excellent (n = 5) work ability. The average age decreases from 52 years in the poor work ability level group to 38 years in the group with excellent work ability level. The average WAI score was 30 with a range of 7–49.

**Table 1:** Mean values and standard deviation of the different test results of physical capacity and mood measurements presented by work ability level

**Work ability level (score range)**	**N**	**Age**	Hand grip strength, kg*	Pinch key, kg*	Pinch 3-chuck, kg*	**Purdue Pegboard^®^**	**HADS D**	**HADS A**
Poor (7–27)	14	52 (10)	36 (18)	8.5 (3.2)	7.9 (3.0)	11 (2.1)	7.0 (5.2)	8.1 (5.9)
Moderate (28–36)	14	50 (14)	44 (17)	10 (2.6)	8.8 (2.6)	13 (2.6)	2.2 (2.1)	4.9 (3.6)
Good (37–43)	14	48 (11)	45 (15)	9.9 (3.0)	8.7 (2.7)	13 (1.9)	2.4 (2.1)	3.5 (1.6)
Excellent (44–49)	5	38 (11)	46 (11)	10 (3.3)	10 (2.4)	14 (1.2)	1.6 (1.3)	2.4 (2.4)

(*Measurements recorded in kg, explained in Methods section).

Table 2 presents sensorineural (stage 0SN-3SN) and vascular (stage 0–4) staging of the dominant hand according to the SWS [7,22]. Thirty-seven subjects were classified with stage 2 or 3 concerning either, or both, sensorineural or vascular symptoms and signs. Vibration white finger was reported by 24 subjects. The table illustrates how the staging is distributed among the workers and that the majority is suffering from a moderate or severe condition.

**Table 2:** **Staging of symptoms and signs in the dominant hand according to the Stockholm Workshop Scale**[14]

**Vascular staging**	** Sensorineural staging**				
	**0 SN**	**1 SN**	**2 SN**	**3 SN**	**Total**
**0**	0	9	11	3	23
**1**	0	1	5	0	6
**2**	1	1	5	1	8
**3**	4	0	5	1	10
**4**	0	0	0	0	0
**Total**	5	11	26	5	47

**Figure 1:**
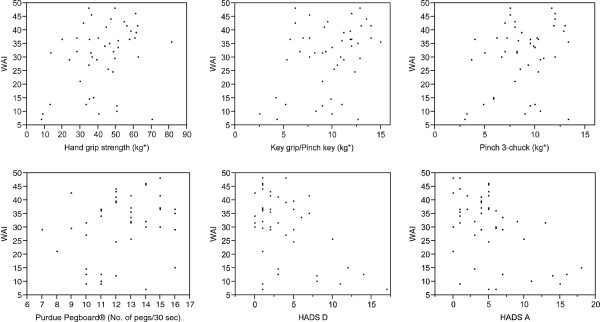
**Correlation plots of Work Ability Index (WAI) versus: hand grip strength, dominant hand; manual dexterity, measured using the Purdue Pegboard^®^, dominant hand; key grip/pinch key, dominant hand; pinch 3-chuck (three-digit pinch), dominant hand; HADS A and HADS D (Hospital Anxiety and Depression Scale; A = Anxiety; D = Depression).** (*units in kg, explained in the Methods section).

Scatter plots were created for the different tests and the WAI (Figure 1). Pearson’s correlation analysis showed statistically significant associations between the WAI and the results of all the quantitative tests (hand-grip strength, key pinch/key grip and manual dexterity using the Purdue Pegboard^®^ for the dominant hand (Table 3), except the Pinch 3-chuck. The WAI was also significantly associated with both HADS indices (Table 4).

**Table 3:** Pearson correlation coefficients for the association between each quantitative test and Work Ability Index

**Variable**	**Mean value (SD)**	**Pearson correlation coefficient (r) and 95% CI**	**p-value**
Hand grip strength (kg)	42 (16)	0.32 (0.028 – 0.56)	0.033
Key grip/Pinch key (kg)	9.6 (3.0)	0.33 (0.049 – 0.57)	0.023
Pinch 3-chuck (kg)	8.6 (2.8)	0.27 (−0.017 – 0.52)	0.064
Purdue Pegboard^®^	12 (2.2)	0.32 (0.029 – 0.56)	0.032

SD = standard deviation.

**Table 4:** Pearson correlation coefficients for the association between HADS, age and Work Ability Index

**Variable**	**Mean value (SD)**	**Pearson correlation coefficient (r) and 95% CI**	**p-value**
HADS A^a^	5.2 (4.4)	−0.41 (−0.64 - -0.11)	0.007
HADS D^a^	3.7 (4.0)	−0.42 (−0.65 - -0.12)	0.007
Age	49 (12)	−0.33 (−0.57 - -0.04)	0.028

*HADS* = Hospital Anxiety and Depression Scale; *A* = Anxiety index; *D* = Depression index.

^a^ Log transformation for the HADS was used.

When analyzing the male workers separately the correlation coefficients between WAI and the different tests did not differ much except for the correlation coefficient of Purdue Pegboard^®^ that was decreased and the p-value was above 0.05. The sample of women was not analyzed separately due to the small sample size.

The statistically significant variables (p ≤ 0.05) from the different tests (hand grip strength, pinch key and the HADS D index) were used in a multiple regression model which also included age and the aforementioned dichotomized SWS variable. The HADS D was selected to represent the influence of psychological mood; however, substituting HADS A, the resulting model was similar. Using the backward selection method, a significant model emerged (F = 15.5; p < 0.0001; R square = 0.418.) Significant variables are presented in Table 5. Neither the dichotomized SWS variable nor the results from the quantitative tests were statistically significant predictors of work ability.

**Table 5:** Predictor variables for Work Ability Index

**Predictor variable**	**Estimate**	**P**
Age	−0.38	0.0021
HADS D index	−1.72	<.0001

*HADS D* = Hospital Anxiety and Depression Scale, Depression index.

The resulting regression equation was:

$$\text{WAI}=55.2-\left(0.38\times \text{age}\right)-\left(1.72\times \text{HADS D}\right)$$

## Discussion

The results from the explorative correlation analyses in the present study indicate statistically significant associations between work ability and the different quantitative tests on physical capacity as well as HADS (depression and anxiety) questionnaire results. Further analyses using multiple regression models suggest that the results from tests on physical capacity and sensorineural/vascular staging are not statistically significant predictors of work ability, but that psychological mood, whether assessed as depression or as anxiety, is a statistically significant predictor of work ability. According to the overall resulting model, approximately 42% of the variation in WAI is explained by age and psychological mood.

Age has previously been shown to affect work ability, especially in physically demanding jobs [23], though for men performing mental work, there was no systematic decline in the index up to the age of 57 years. The authors of that study explained this by a decline in physical capacity due to “no participation in regular physical training”. This could be a plausible explanation but as age increases, so may the effects from work and other exposures increase that can affect health and functioning and, consequently work ability. The associations between WAI and the test results from measuring hand grip strength, finger strength and manual dexterity seem to be apparent because these values change with age. This may indicate that the effect from vibration exposure is not severe enough, that the affected individuals do not subjectively consider themselves affected or that conditions mainly affecting the hands are not sufficiently recognized in the WAI.

Other studies [10,11] have reported similar relations to those found in the present study with dexterity and hand grip-strength correlated to dysfunction (in terms of the DASH score and ADL). Cederlund et al. report statistically significant correlations between (dominant hand) hand grip strength and manual dexterity (measured using the Purdue Pegboard^®^) and difficulties in ADL performance, and conclude that difficulties in managing daily activities are commonly seen among vibration-exposed workers. In comparison to ADL during leisure time, work ability may be spurred by a stronger drive or may depend on other aspects of motivation [24]. It is therefore important to consider not only the individual’s physical capacity but also their psychological symptoms, which seem to have a significant impact on WAI. A worker with an injury or disease in the hands or fingers resulting in physical dysfunction must not only be given proper treatment to avoid any further dysfunction, but must also receive appropriate caretaking for any psychological symptoms, in order to minimize their suffering and restore as much as possible of their potential work ability. This can raise questions as to whether the self-reports of work ability using the WAI adequately reflect a potential decline in physical capacity; it is possible that the questionnaire may put too much focus on the psychological mood. However, this consideration is in contrast to a previous study [25] on the association between functional capacity and work ability where the authors claim with some certainty that “the used work ability index measured primarily physical features of work ability”. Their methods of measuring “mental capacity”, however, involved using tests of visuomotor speed (measured using the digit symbol test) and perceptual and conceptual ability, and not questionnaires.

The average WAI score of 30, in this sample of workers with HAVS, falls in the category of moderate (27–36) work ability, which can be considered, in comparison, as quite low. There are few studies reporting on mean WAI score among blue collar workers; however, de Zwart et al. [26] investigated the reliability of WAI among construction workers (aged 40 years and above) with a result of WAI around 40, which belongs to the category of good work ability. A study on Finnish dairy farmers [27] reported a mean WAI score of 36 among female workers and 39 among male workers. The difference between the current WAI score and the previously reported may be explained by the participation of former patients with existing symptoms in the current study. There is also a possibility of further decrease in mean WAI score due to psychological mood disorders as a result of the hand symptoms and disability.

The present study is an attempt to explore the possibility of creating a model that can predict work ability to a sufficient extent. The associations between WAI and test results from measuring hand grip strength, finger strength and manual dexterity seem to be apparent because these values change with age. Accordingly, the test results may be difficult to use as a direct instrument for assessing work ability but may still have a role as a complementary measurement in the clinical examination and assessment. However, the results reflects measurements from a limited group of hand arm vibration-exposed patients and therefore need to be interpreted with caution and confirmed in larger studies. The physical capacity in terms of measurements of hand and finger strength and manual dexterity may also be too limited or specific which can raise the question of whether another measure or other measures of physical capacity would be more relevant in explaining the level of work ability in these patients.

This study, however based on a small sample and exploratory, may hopefully offer some insight into how to advice the clinician when assessing patients with HAVS. The clinician may use the WAI and the psychological test as a complement to the more established tests of physical capacity in the hand in order to achieve a more general picture of the workers’ health. This can be especially important if there is not a clear relationship between staging of symptoms/signs of vibration injury and work ability, as indicated by the multiple regression model. Furthermore, the results from psychological testing may show that the psychological mood of patients with hand symptoms exposed to vibrating tools must be taken into deeper consideration.

The challenge is to provide adequate guidelines with a sound basis when more objective assessments are needed, for instance when issuing physicians’ (sick-leave) certificates and when assessing patients for rehabilitation or financial compensation. In order to make these assessment systems fair and appropriate, there is a need for objective methods which can be used instead of subjective assessments. It is not sufficient to focus on the staging of symptoms and signs in these affected workers. The SWS, though criticized for its shortcomings, is used (in some countries) for decisions on financial compensation and also to determine fitness for work [28]. This may be misleading and can result in incorrect conclusions. There is a need for improvement of the communication and understanding between different operators (e.g. occupational/primary health care services, employers, employees and the social insurance agency) on this issue [29,30]. Hopefully some new perspective on this complex condition of vibration injury can contribute to an improvement in assessment systems.

## Conclusions

Age and psychological mood seem to be stronger predictors of work ability than results from tests measuring physical capacity in vibration-exposed patients with hand symptoms. When using the WAI as an instrument for assessing work ability in these patients, health care providers need to be more aware of the impact of the psychological mood.

## Competing interests

The authors declare that they have no competing interests.

## Authors' contributions

ME wrote the manuscript, contributed to the design of outcome measurements, performed the statistical analyses and the interpretation of data. MH and LG initiated and designed the study, discussed and contributed to the manuscript and the interpretation of data. All authors read and approved the final manuscript.
